# Progressive Macular Hypomelanosis: A Rarely Diagnosed Hypopigmentation in Caucasians

**DOI:** 10.1155/2009/607682

**Published:** 2009-06-01

**Authors:** Sven Neynaber, Christina Kirschner, Stefanie Kamann, Gerd Plewig, Michael J. Flaig

**Affiliations:** Department of Dermatology and Allergology, Ludwig-Maximilians University, Frauenlobstrasse 9-11, DE-80337 Munich, Germany

## Abstract

A 35-year-old woman who developed whitish macules on trunk and limbs at 12 years of age and observed a remarkable increase of the hypopigmentated lesions after her pregnancies at ages 29 and 32 years. Because of the highly characteristic clinical aspect and the light- and electron-microscopic histopathologic findings, we diagnosed progressive macular hypomelanosis (PMH). It is a nonscaly disorder with hypopigmented macules mainly on the trunk and is more often seen in young women. In contrast to some authors assuming the presence of *Propionibacterium spp.* as a matter of principle in PMH, we report a case with no evidence for *Propionibacterium spp.*

## 1. Introduction

Hypopigmented lesions of the skin are common and display in general no diagnostic challenge. In Caucasians, in particular in persons with light skin type I to III according to Fitzpatrick [1], progressive macular hypomelanosis (PMH) is a rarely diagnosed disease posing a relevant differential diagnosis for hypopigmentations. It is a primary, acquired hypopigmentation first described by Guillet et al. [2] affecting mainly women 18 to 25 years of age. We here report on a female Caucasian patient with PMH living in Germany who observed an increase in hypopigmentation after pregnancies. 

## 2. Case Report

A 35-year-old woman with skin type III was referred to our department for the evaluation of whitish macules on trunk and limbs. They first appeared when she was 12 years old and increased slowly but steadily in size and number. After the first gravidity at age 29 and a second one at age 32 the patient observed a marked acceleration of this process. There is no history of itching or dysaesthesia. The patient denied recreational sun exposure for the past ten years. When she was exposed to sunlight the whitish macules did not change the colour whereas the nonaffected skin became more tanned, so that the contrast between the whitish macules and the nonaffected skin was remarkably intensified. She had not been on antibiotics, birth-control or hormone, containing pills, had no noteworthy history of dermatitis or eczema. Signs of atopy were absent. Her ancestors lived for many generations in Germany. 

**Figure 1:**
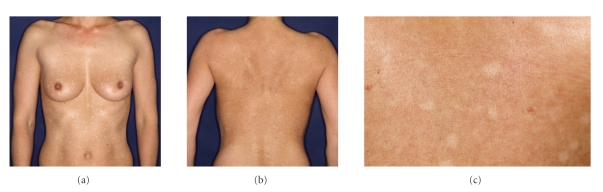
(a) Disseminated whitish macules on the front and the back of the trunk. (b) Nonscaly macules in detail on the back.

Skin findings consist of symmetrically distributed hypopigmented, nummular, partial confluent, nonscaly macules on trunk and less pronounced on proximal parts of arms and legs, as well as sparing head and neck (Figures 1(a), 1(b)). Distribution is neither segmental nor does it follow Blaschko-lines. The round or oval hypopigmentated macules have well-defined borders. No signs of inflammation are present in these macules. Uninvolved skin displays brownish coloration alike typical skin type III. 

There was no evidence for fungi or other infectious agents in morphologic and cultural surveys on repeated attempts. A Biopsy from affected skin displayed a reduced amount of melanin in the basal layer compared to nonaffected skin. 

**Figure 2:**
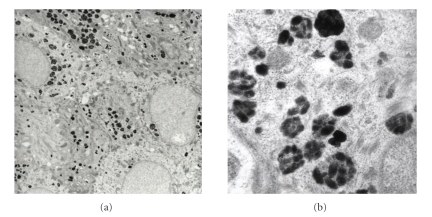
Electron microscopy of *non-affected skin*. Left (8:300×): numerous melanosomes aggregated in big clusters and disseminated melanosomes. Right detail (49:800×): Granular melanosomes mostly grade III-IV.

**Figure 3:**
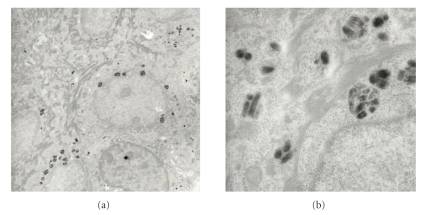
Electron microscopy of *affected skin*. Left (8:300×): sparse melanosomes aggregated in small clusters and some solitary. Right detail (49:800×): Melanosomes mostly grade I-II, few grade III and less granular.

Upon electron microscopic comparison of type and distribution patterns of melanosomes in affected (Figure 3) and nonaffected epidermis (Figure 2), we found those in macules of PMH to be more aggregated and single melanosomes were smaller. They displayed filaments and looked tessellated (stage I-II). In contrast the melanosomes in nonaffected skin are usually larger and distributed more evenly with less discernible internal structures. In other words the affected skin is characterized by less developed melanosomes (stage I or II), whereas nonaffected epidermis displays mature melanosomes (stage III or IV) according to the natural skin type of the patient.

## 3. Discussion

PMH was first published by Guillet et al. in 1988 [2]. He described a disorder prevalent in young women (aged 13 to 35 years) in the West Indies and in a Caribbean immigrant population in France characterized by spreading hypochromic macules on the trunk. The condition appears mainly in persons of mixed genetic background [3] with round, pale, coalescent macules on the back and less frequently on the abdomen. A larger study performed in Martinique revealed the condition to be more widespread in men than in women, with a mean age of 25 years [4]. PMH is until recently thought to be an idiopathic disease wich is rarely seen in middle and northern Europe, being much more frequently reported from countries with a multiethnic population of skin type IV–VI [1]. This case seems to be the first reported patient with PMH in Germany. Recently Di Lernia et al. [5] reported five patients in Italy of skin type IV-V, three of them of Caucasian one of North-African, and one of Afro-Caribbean origin, respectively. Kumarasinghe et al. [6] reported on eight Chinese patients with PMH. 

Several differentials have to be ruled out once PMH is considered: Pityriasis alba is a benign condition in children and adolescents, which is frequently located on the face presenting a hypopigmentation with fine pityriasiform scaling. Postinflammatory hypopigmentation is usually more localized than PMH and occurs in the aftermath of inflammatory conditions such as lichen planus or eczema as irregularlly shaped lesions. Idiopathic guttate hypomelanosis is known as a disorder of both sexes going along with multiple tiny confetti-like hypopigmented macules on extremities, especially on calves and shins, representing, therefore, the closest differential diagnosis to PMH. 

In general PMH does not respond well to antifungal or antieczematous therapy. A recent study of 45 patients with intraperson comparison of two treatment strategies—5% benzoyl peroxide hydrogel/1% clindamycin lotion in combination with UVA irradiation versus 0.05% fluticasone propionate cream in combination with UVA irradiation—found the antibacterial treatment superior (photometric measurements *P* = .007, patient assessment *P* < .0001, and dermatologist assessment *P* < .0001) [7]. PUVA und UVB therapy was reported to achieve improvement [8], at least transiently in a few cases. Anyhow, sometimes the PMH regresses spontaneously within a few years.

A cosmetic concern is frequently the reason for consultation of a dermatologist because the young women feel cosmetically deprived with in times psychological repercussions. Authors around the globe used terms like cutis trunci variata [9], creole dyschromia [4], idiopathic multiple large-macule hypomelanosis [10], and nummular and confluent hypomelanosis of the trunk describing similar symptoms as in PMH. 

In our patient we did not find an evidence for fungi, neither in cyanoacrylate surface skin biopsies nor morphologically in KOH preparations of scrapings, nor in culture, nor histologically in the biopsy. These negative mycological investigations support the findings from Westerhof et al. [11] and Guillet et al. [4] that fungi are not likely causal to PMH. 

Relyveld et al. postulate that different subtypes of *Propionibacterium species* cause the appearance of PMH [12]. But species of *Propionibacteria* can be found all over the body and are generally nonpathogenic. To the best of our knowledge there is no further evidence in the English and German literature available in support of a pathogenic role of *P. acnes ssp*. in hypopigmentation. This finding has to be verified independently in the future. On multiple attempts we did not find any evidence for this pathogen in this patient, neither clinical with Wood's light, nor histological in special stains (Gram, Giemsa). Therefore we question the causality of *Propionibacteria in* PMH, as, for example, many patients suffering of acne do have massive *Propionibacteria* colonisation and do not show hypopigmentation as in PMH, nor is hypopigmentation on face and other affected areas a typical feature in these patients. 

With electron microscopy investigations we confirmed a distinct change in the maturation, number, and distribution of the melanosomes in the affected skin. Theses results are consistent with those of Relyveld et al. [12, 13], although PMH is more often diagnosed in skin type IV-V with electron microscopy findings beeing more distinctive. 

The accelerated progression of hypopigmentation observed here during pregnancy could suggest that hormones may play a pathogenetic role albeit serological misbalance of hormones was not found during gestation. There are many skin disorders known to be worsened through pregnancy and it could be the same with PMH. Another clue to hormonal influence is that all affected patients in literature were in a reproductive age, whereas the putative gynecotropism could be not solely due to a result of bias in reporting [6], but valid epidemiologic data are not available [6]. In consequence, PMH is a rarely reported entity in Europe whose aetiology still remains an enigma. The clinical course is unpredictable, but spontaneous resolution can occur within years or decades.
